# Southeast Asia Strategic Multilateral Dialogue on Biosecurity

**DOI:** 10.3201/eid2505.181659

**Published:** 2019-05

**Authors:** Anita Cicero, Diane Meyer, Matthew P. Shearer, Sazaly AbuBakar, Ken Bernard, W. Seth Carus, Chee Kheong Chong, Julie Fischer, Noreen Hynes, Tom Inglesby, Chong Guan Kwa, Irma Makalinao, Tikki Pangestu, Ratna Sitompul, Amin Soebandrio, Pratiwi Sudarmono, Daniel Tjen, Suwit Wibulpolprasert, Zalini Yunus

**Affiliations:** Johns Hopkins Center for Health Security, Baltimore, Maryland, USA (A. Cicero, D. Meyer, M.P. Shearer, T. Inglesby);; Johns Hopkins Bloomberg School of Public Health, Baltimore (A. Cicero, D. Meyer, M.P. Shearer, T. Inglesby);; University of Malaya, Kuala Lumpur, Malaysia (S. AbuBakar);; US Public Health Service, Washington, DC, USA (K. Bernard); National Defense University, Washington, District of Columbia (W.S. Carus);; Ministry of Health, Kuala Lumpur (C.K. Chong);; Georgetown University Medical Center, Washington (J. Fischer);; Johns Hopkins University, Baltimore (N. Hynes);; S. Rajaratnam School of International Studies, Singapore (C.G. Kwa);; University of the Philippines Manila College of Medicine, Manila, the Philippines (I. Makalinao);; University of Singapore, Singapore (T. Pangestu);; University of Indonesia, Jakarta, Indonesia (R. Sitompul, A. Soebandrio, P. Sudarmono);; Mayapada Healthcare Group, Jakarta (D. Tjen);; Ministry of Public Health, Bangkok, Thailand (S. Wibulpolprasert);; Ministry of Defence, Kuala Lumpur (Z. Yunus)

**Keywords:** public health, policy, Southeast Asia, strategic multilateral dialogue, biosecurity, containment of biohazards, communicable diseases, epidemiology, disease outbreaks, biological warfare agents, epidemics, pandemics, biological warfare agents, bioterrorism and preparedness, international cooperation, biosurveillance, emerging diseases

## Abstract

A strategic multilateral dialogue related to biosecurity risks in Southeast Asia, established in 2014, now includes participants from Singapore, Malaysia, Indonesia, Thailand, Philippines, and the United States. This dialogue is conducted at the nonministerial level, enabling participants to engage without the constraints of operating in their official capacities. Participants reflect on mechanisms to detect, mitigate, and respond to biosecurity risks and highlight biosecurity issues for national leadership. Participants have also identified factors to improve regional and global biosecurity, including improved engagement and collaboration across relevant ministries and agencies, sustainable funding for biosecurity programs, enhanced information sharing for communicable diseases, and increased engagement in international biosecurity forums.

A Strategic Multilateral Dialogue on Biosecurity was established in 2014 that now includes participants from Singapore, Malaysia, Indonesia, Thailand, the Philippines, and the United States. This dialogue was initiated to engage high-level current and former government officials and nongovernmental experts and stakeholders in candid discussions about the priorities, challenges, and developments related to biosecurity risks in Southeast Asia.

This biosecurity dialogue is conducted at the Track II level, rather than as a formal Track I (i.e., ministerial level) exchange. Track II diplomacy involves informal discussions that take place outside official government channels, enabling a more open and frank exchange of ideas and exploration of possible solutions to problems (*1*). These types of dialogues provide a forum for participants to establish lasting relationships and partnerships with their counterparts in other countries in advance of a crisis and to discuss common challenges and best practices. Track II formats enable participants to freely share perceptions, vulnerabilities, and new ideas without the constraints experienced when persons are operating in their respective official capacities.

Participants in this dialogue (Appendix), which includes up to 5 participants from each country, reflected collectively about how to effectively detect and mitigate biosecurity risks, especially those faced in Southeast Asia, and they shared information and exchanged ideas on mechanisms to highlight major biosecurity issues for national leadership. Participants included current and former senior-level government officials and subject matter experts from across relevant ministries and sectors, including academia, health, defense, and homeland security/home affairs. Persons who have participated in this dialogue since its inception are also listed (Appendix). These participants are also in positions to transmit key messages to the most senior levels of government and through major regional forums, such as the Association of Southeast Asian Nations (ASEAN). The Johns Hopkins Center for Health Security convenes the dialogue and has hosted meetings thus far in Singapore, Malaysia, Indonesia, and the United States. The purpose of this report is to describe insights that have emerged from this ongoing dialogue and suggest priority areas for action to strengthen national and regional biosecurity.

## Biological Threats that Can Impact Regional and Global Health, Security, and Economies

Numerous risk factors in Southeast Asia increase the vulnerability of this region to natural, deliberate, and accidental biological threats. Countries in the region have made major progress in fighting infectious diseases within their own borders; however, the widespread geographic population distribution—ranging from remote, rural villages to densely populated cities—combined with highly mobile populations (e.g., tourists, migrant workers, displaced persons) and areas of porous international borders create a dynamic human–animal–plant–environment (i.e., One Health) interface that enhances the susceptibility of the region to the emergence and spread of infectious diseases. Because of increasing ease and speed of travel, infectious disease threats in a country are threats to neighboring and distant countries alike. In Southeast Asia, intraregional airlines have seen explosive growth over the past 10–15 years, making regional travel more convenient and affordable (*2*–*4*). The susceptibility of this region to the spread of infectious diseases was exemplified during the severe acute respiratory syndrome (SARS) epidemic during 2003, which began with the index case in Guangdong Province, China, and rapidly spread around the world, including to Indonesia, Malaysia, the Philippines, and Singapore, ultimately killing >750 persons (*5*).

In addition to the cost of human lives, infectious disease outbreaks can seriously damage or even devastate economies because of impacts on international trade and local tourism, both of which might slow or grind to a halt. Again, when we used the SARS epidemic during 2003 as an example, Malaysia lost ≈US $1.7 billion and Thailand lost ≈US $33.5 billion in tourism, and Singapore lost ≈US $4.9 billion in gross domestic product during 2003 (*5*). A dialogue participant also discussed the tremendous economic impact of the outbreak of Nipah virus infection in Malaysia during 1998–1999, which resulted in the culling of >1 million pigs, essentially shuttering the pork industry of this nation and exerting a tremendous economic toll (*6*).

Even events that do not result in any local cases can have high financial impact. For example, during the 2013–2016 West Africa Ebola epidemic, countries in Southeast Asia spent millions of dollars on Ebola preparedness, and some countries, such as Malaysia, mobilized medical and public health resources to support the response in West Africa. Countries in the Southeast region, including Thailand and Indonesia, used precious resources to develop and disseminate Ebola preparedness plans, conduct risk assessments to evaluate the likelihood of introduction of Ebola, increase operational readiness to isolate and manage suspected cases, and prepare Ebola-specific risk communication (*7*).

Growing threats from sophisticated terrorist groups, including a number emerging from Southeast Asia, have raised concerns about the potential for deliberate biological attacks in the region and across the globe. A particularly concerning trend is the increasing presence of the so-called Islamic State and affiliated groups, as territorial losses in Iraq and Syria have forced them to shift operations to other regions (*8*). In addition, an increasing number of high-containment laboratories that work with high-consequence human, animal, or plant pathogens and emerging biotechnology, including synthetic biology, highlight the need for structured laboratory oversight to prevent deliberate and accidental releases of dangerous pathogens. Detection and prevention of deliberate misuse will be challenging because of the increase in dual-use research (i.e., life sciences research with the potential to be used both for beneficent and nefarious purposes) (*9*). It is simply not possible to eliminate the risk of the nefarious use of biology because of the ubiquity of raw materials, equipment, basic scientific knowledge, and advanced technologies. Thus, efforts to engage the scientific community and implement effective organizational oversight and rigorous monitoring and reporting procedures are critical to mitigating the risk. If one considers the potential consequences to public health, national security, and the economy, dedicated national, regional, and global efforts are required to prepare for and respond to a broad range of biologic threats, whether they be natural, accidental, or deliberate.

## Preparing for and Responding to Biological Threats Requires Coordination across Disciplines and Sectors

The complex nature of biosecurity threats necessitates multisectoral engagement to identify and implement effective prevention and response mechanisms. These mechanisms cannot be siloed as the sole responsibility of any ministry or agency. Although the most likely high-impact biological threats continue to be emerging or reemerging pathogens, deliberate biological events and high-consequence accidents are also increasingly possible. Preparedness and response activities to mitigate the risk and effects of biological threats should involve representatives from the public health and healthcare, defense, home affairs/homeland security, agriculture, and trade and finance sectors, in addition to other relevant government agencies. To ensure that any plans and policies are practical and acceptable and address the needs of diverse communities and populations, inputs from academia, nongovernmental organizations, regional organizations, the media, and other parts of civil society should also be considered in biosecurity planning and preparedness efforts.

Collaboration outside the human health sector, including animal, plant, and environmental health, is particularly needed for mitigating the spread of life-threatening infectious diseases and manage the threat of bioterrorism. A dialogue participant discussed how the first identified the outbreak of Nipah virus infection in Malaysia (1998–1999) could have been much worse. Malaysia was able to integrate human and animal health (i.e., One Health) in the midst of the response, but the outbreak emphasized the need to structure the collaboration in advance of events to improve response capacity. Another participant discussed how the veterinary field epidemiology training program in Thailand promotes information sharing. This program includes human and animal health professionals, and the relationships formed helped improve informal collaboration through this network, even if formal mechanisms do not exist. Similarly, Malaysia encourages public health students to participate in veterinary field work to develop cross-sectoral expertise and relationships. Collaborative preparedness efforts across sectors before an event will help to facilitate successful joint preparedness and response efforts.

Responding to biosecurity events will also require integration of national security, domestic intelligence, and law enforcement into public health and healthcare preparedness and response efforts. Information and intelligence sharing between these disciplines will be critical because data about the pathogen and exposure will be necessary to support the public health response, including development or selection of medical countermeasures and personal protective equipment, as well as for forensics and attribution efforts. For example, conducting joint training and exercises could initiate a foundational relationship between sectors and streamline responsibilities to prevent duplication of efforts. Collaboration among the health, including human, animal, and plant, and security sectors could also help protect military and law enforcement responders and align security and logistics support with activities to protect the public’s health, including medical countermeasure distribution and dispensing. Dialogue participants from Malaysia noted that their government has identified a need to strengthen coordination between public health agencies and law enforcement, and they have implemented joint training programs to facilitate effective collaboration, specifically for deliberate biological event responses and investigations. Malaysian law enforcement also supports laboratory personnel reliability programs and seeks to better understand the types of research going on throughout the country. Furthermore, Malaysia is in the process of approving national legislation to enable the country to meet nonproliferation obligations under the Biological and Toxin Weapons Convention (BWC) and to inhibit and interdict those who seek to misuse the life sciences.

Targeted and scheduled training with all relevant agencies involved in preventing, responding to, and mitigating biological threats is essential to enhance multisectoral coordination and communication. Cross-agency training is also needed for risk communication, which is currently not given sufficient emphasis. Risk communication planning should be prioritized across relevant agencies because effective communication will assist in efforts to inform the affected population, encourage the adoption of appropriate protective behavior, and limit the impact of adverse events during a biological incident. Improved training, tools, and resources are needed to help health communicators, first responders, and response leadership communicate effectively during a biological incident. Critically, pre-event collaboration with ministries of economy, finance, and commerce could help governments better forecast and mitigate the economic effects of biological events. The financial consequences of biological threats are often neglected during biosecurity planning, preparedness, and response activities. As noted earlier, biological events can result in major costs and economic losses for affected countries and regions well beyond the direct costs of preparedness and response activities (*10*,*11*). In addition, many countries recognize advances in biotechnology as tools for economic growth. However, as a participant from Singapore noted, the Ministry of Trade has oversight authority for genetic modification, but trade officials often view these capabilities through an economic lens without necessarily recognizing the potential risks associated with certain types of research. Considering these potential impacts, collaboration among health officials; economics experts; industry (including the tourism sector); health, safety, and security agencies; and elected officials would provide the diverse perspectives necessary to more adequately anticipate the financial and health impact of biosecurity events.

## Necessity of Sustained, Reliable Funding to Address Biosecurity Challenges

Many countries face challenges in sustaining consistent and reliable funding for biological preparedness and biosecurity. In the time between infectious disease outbreaks or other biological events, decision-makers can become complacent, resulting in decreased interest in and awareness of biological threats, often accompanied by fluctuations in funding for preparedness and response efforts. When preparedness and biosecurity programs succeed in preventing or mitigating the effects of biological events, funding may be paradoxically reduced because of perceptions that threats have been adequately addressed and no longer require investment. In addition, meaningful metrics for measuring success in preventing a consequential biological event are virtually impossible to create, further contributing to difficulties in advocating for robust funding.

After initial investments have been secured for the development and implementation of national biosecurity structures and programs, sustained and reliable funding and resources are necessary to maintain them, as noted by multiple dialogue participants. In the time between outbreaks and other biosecurity events, continual training and stable funding are required to ensure personnel maintain proficiency, equipment is properly updated and maintained, and supplies (e.g., personal protective equipment, medical countermeasure stockpiles) are periodically replaced. A participant noted the challenge of sustaining biosecurity funding in the face of other unconventional weapons threats, referencing a shift of national funding away from biosecurity and toward chemical and nuclear threats because of concerns about recent events in other countries, including nuclear weapons negotiations in North Korea and chemical weapons use in Syria and the United Kingdom. Without long-term investments in organizational structures and biosecurity programs, skills and materiel stagnate and response capabilities degrade, eventually requiring larger investments during future public health emergencies to bolster atrophied capacity. Effective biosecurity programs require dedicated long-term support as opposed to 1-time emergency resource allocations in response to individual events.

Biosecurity programs are necessarily spread across many agencies and ministries, and the role these programs play might not be readily identified or appreciated by officials outside health and national security sectors. For example, a participant noted that laboratory equipment for healthcare or public health research may also be used to support biosecurity incident response. It is critical to inform policymakers of the effects of investments in science, healthcare, public health, national security, and other sectors and how they serve to support preparedness and response efforts for biosecurity threats, so that they can truly understand the value of these investments. For example, when the Thailand Ministry of Health initially proposed a major investment in domestic influenza vaccine production capacity in 2006, the idea received pushback in light of existing global production capacity. When health officials highlighted the national security threats that global production shortages during a pandemic could pose, the proposal ultimately garnered support from the Ministry of Defence.

Framing biological threats from an economic security perspective, rather than solely in terms of potential human health impacts (i.e., illness and death), is another option to help nonhealth officials more fully appreciate the value of biosecurity preparedness investments. For instance, some participants noted that it might be useful to calculate estimated financial losses that could result from an infectious disease outbreak to inform decisions regarding investments in establishing or maintaining biosecurity programs at the national, state, and local levels as well as allocation of emergency funding in preparation for or in response to a biosecurity event.

## Improved Preparedness through Formal Arrangements for Sharing Regional Information about Outbreaks and Other Biological Threats

As the biosecurity landscape grows more complex because of emerging and reemerging infectious diseases, increasingly mobile populations, and advancing technologies, there is an increased need for countries to work together to prevent, detect, and respond to biological threats. Infectious diseases do not respect borders, and biological events in a country can quickly spread to neighboring countries and around the world.

High-quality, timely data are necessary for decision-makers to direct response activities. Well-designed and effective surveillance systems are critical for identifying the emergence of biological events, but most systems are not designed to facilitate information- and data-sharing between government agencies or between different countries. Without these connections, it might be difficult to identify an emerging outbreak, particularly if cases are spread across several countries.

A participant from Thailand commented that the Mekong Basin Disease Surveillance Regional Network was critical to supporting the response to a case of influenza A(H5N1) imported from Laos to Thailand during 2005. It was noted that these 2 countries are in different World Health Organization (WHO) regions (Western Pacific for Laos and Southeast Asia for Thailand) and that responders used personal relationships through the Mekong Basin Network because coordination through WHO proved difficult. Information sharing between countries, especially those within the same geographic region, is key to ensuring that outbreaks are quickly identified, characterized, and reported. Increased information sharing could also result in a common operational picture with respect to outbreaks and other biological events in the region. Currently, data and sample sharing in Southeast Asia is often conducted on an ad hoc basis for individual events and based on personal relationships between colleagues across borders, rather than through official government engagements and formal agreements. Mechanisms for more formal and regularized information sharing should be explored, perhaps regionally through ASEAN or other international or regional forums. A participant noted that the emergence of SARS during 2003 led to increased attention to biosecurity in the ASEAN regional forum; however, funding and support for these programs have decreased in recent years, illustrating the need to prioritize regional collaboration on biosecurity issues and increase awareness of regional threats. The need for this kind of exchange should be raised to the attention of senior government officials who have the authority and responsibility to propose and negotiate formal regional and international mechanisms to share disease data and financial support for regional biosecurity programs, with the aim of improving event prediction and detection and speeding response activities.

## Improvement in Preparedness for Biosecurity Threats through Increased Commitment to International Agreements

Another fruitful way for countries to improve their biosecurity and biorisk management capabilities over time is through engagement in international mechanisms that reinforce biopreparedness norms and bolster international linkages that result in the sharing of best practices. The key requirements for countries to strengthen their biosecurity capabilities are well articulated in the WHO International Health Regulations core capacities. Launched in 2014, the Global Health Security Agenda (GHSA) now has >64 partnership countries (*12*), and it has been previously chaired by several countries in Asia, including Indonesia and South Korea. The GHSA underscores the need for a multilateral and multisectoral approach to strengthening national capacities of countries to prevent, detect, and respond to naturally occurring, accidental, and deliberate infectious disease threats (*12*). It is designed to help countries obtain support, including funding and training, in their efforts to meet commitments under the International Health Regulations (*13*), the World Organisation for Animal Health Performance of Veterinary Services pathway (*14*), and other relevant global frameworks. A participant from Indonesia discussed the role of Indonesia as Chair of the GHSA Steering Group during 2016 in increasing awareness of biosafety and biosecurity challenges among national government officials. The GHSA has proved to be a powerful tool for assessing national capacity and identifying and addressing global gaps in health security in advance of future health emergencies.

Other international agreements, such as the BWC (all dialogue counties are states parties to the BWC) and United Nations Security Council Resolution 1540, aim to prevent the deliberate misuse of biology (e.g., biological weapons). However, these agreements also serve as mechanisms to share biosecurity and biosafety best practices internationally and obtain assistance in developing and implementing national-level biosecurity programs and policies. Many countries obtain some technical support and capacity building through the BWC; however, several participants noted that some countries are not always able to invest the resources necessary to effectively engage in critical global policy issues. Continued engagement in the BWC and similar international forums not only serves to bolster international bioweapons nonproliferation norms, biosafety, and biosecurity but also supports countries in building domestic capacity to predict, prevent, detect, and respond to biological events from any cause or source.

## Conclusions

Track II exchanges facilitate collegial problem solving around shared challenges. The participants in the Strategic Multilateral Dialogue on Biosecurity judge it to be a particularly valuable Track II effort, and they have identified several factors and principles for action that would strengthen biosecurity in the region and beyond.

More substantial national, regional, and global efforts are required to prepare for and respond to biological threats, whether they be natural, accidental, or deliberate. In support of these efforts, engagement and collaboration among government officials across relevant sectors, diplomats, subject matter experts, industry (including the tourism sector), and elected officials would bring range of perspectives needed to anticipate the broad range of impacts, including financial, posed by biological threats. Sustained funding to build and maintain biosecurity programs is needed to make further progress compared with 1-time emergency resource allocations in response to individual events. Regionally, mechanisms for improved information sharing around disease surveillance and reporting should be explored, perhaps through the ASEAN Asia Pacific Strategy for Emergency Diseases or other means, and government leaders should be made aware that this kind of international collaboration is key to improving event detection and speeding response activities. Finally, engagement in the GHSA, BWC, and other international forums related to emerging infectious disease response, biosafety, and biosecurity will help create the necessary momentum to build national capabilities and capacities to prepare for and respond to these threats and improve regional efforts. In summary, these kinds of structured approaches could help countries in Southeast Asia obtain technical and material support and create the necessary momentum to better detect, respond to, and mitigate the full range of biosecurity threats the region might face in the future.

Appendix Participants in the Southeast Asia strategic multilateral dialogue on biosecurity.
