# Supplementary material for: Southeast Asia Strategic Multilateral Dialogue on Biosecurity
Source: Emerg Infect Dis. 2019 May;25(5):e181659. doi: 10.3201/eid2505.181659 (PMC6478199; doi:10.3201/eid2505.181659)
Supplement: Appendix — Participants in the Southeast Asia strategic multilateral dialogue on biosecurity. [file 18-1659-Techapp-s1.pdf]

Article DOI: http://doi.org/10.32301/eid2505.181659

Southeast Asia Strategic Multilateral Dialogue on Biosecurity

Appendix

Dialogue Participants

Sazaly AbuBakar, PhD, FASc, Senior Professor/Director, Tropical Infectious Diseases Research and Education Centre and World Health Organization Collaborating Centre for Arbovirus Reference and Research (Dengue/Severe Dengue), Universiti Malaya, Malaysia; Mely Anthony, PhD, Head, Non-Traditional Security Centre, S. Rajaratnam School of International Studies, Nanyang Technological University, Singapore; Rozanah Asmah Abd Samad, DVM, PhD, Senior Principal Assistant Director, Surveillance and Epidemiologic Section, Biosecurity Management Division, Department of Veterinary Service, Ministry of Agriculture and Agro-Based Industry, Malaysia; Endy M. Bayuni, Editor-in-Chief, The Jakarta Post, Indonesia; Kenneth Bernard, MD, US Public Health Service (Retired), Advisor, National Security and Biodefense, USA; Elizabeth Cameron, PhD; Director, Countering Biologic Threats, White House National Security Council, USA; W. Seth Carus, PhD, Distinguished Professor of National Security Policy and Emeritus, National Defense University, USA; Chen Chaw Min, PhD, Secretary General, Ministry of Health, Malaysia; LTC Jeremiah Chng, MD, MPH, Head, Singapore Armed Forces Biodefense Centre, Singapore; Tawee Chotpitayasunondh, MD, Associate Professor (Honorary) and Senior Medical Officer, Queen Sirikit National Institute of Child Health, Thailand; Teck-Mean Chua, DMD, Consultant, Biosciences, Temasek Lifesciences Laboratory, Singapore; Anita Cicero, JD, Deputy Director, Johns Hopkins Center for Health Security, USA; Richard Danzig, JD, PhD, Member, Defense Policy Board, President’s Intelligence Advisory Board; Chairman, Center for a New American Security; Former Secretary, US Navy; Rolando Enrique Domingo, MD, Undersecretary of Health, Office for Health Regulation, Department of Health, Republic of the Philippines; Bryan D. Edmunds, Lt Col, USAF, Global Futures Office, Office of the Vice Director for Plans and Programs, Defense Threat Reduction Agency, US Department of Defense; Francesco Gaetano Fazzi, Lee Kuan Yew School of Public Policy, National University of Singapore; Julie E. Fischer, PhD, Co-Director,

Center for Global Health Science and Security, Georgetown University Medical Center, USA; Gigi Gronvall, PhD, Senior Scholar, Johns Hopkins Center for, Health Security, USA; D. Christian Hassell, PhD, Deputy Assistant Secretary of Defense for Chemical and Biologic Defense, US Department of Defense; Derek Ho Yeong Thye, MSc, Director-General, Environmental Public Health Division, National Environmental Agency, Singapore; Peter Ho, MA, Visiting Scholar, Lee Kuan Yew School of Public Policy; Adjunct Professor, S. Rajaratnam School of International Studies, Nanyang Technological University; Senior Advisor, Centre for Strategic Futures; Former Head of Singapore Civil Service; Former Permanent Secretary for Foreign Affairs, National Security and Intelligence Coordination, Special Duties and Defence, Singapore; MG John P. Horner, Deputy Director, Defense Threat Reduction Agency, US Department of Defense; William P. Hostyn, MS, Director, Advisory Committees and Programs Office, Defense Threat Reduction Agency, US Department of Defense; Noreen A. Hynes, MD, MPH, Associate Professor of Medicine (Infectious Diseases) and Public Health (International Health) and Director, Geographic Medicine Center of the Division of Infectious Diseases, Johns Hopkins University, USA; Tom Inglesby, MD, Director, Johns Hopkins Center for Health Security, USA; Hamzah Ishak, Under Secretary, National Crisis Management and Investigation Division, National Security Council, Prime Ministers Department, Malaysia; Manikavasagam Jegathesan, MD, Former Deputy Director General, Ministry of Health, Malaysia; Barbara Johnson, PhD, RBP, Owner, Biosafety Biosecurity International, USA; CDR Franca R. Jones, MS, PhD, Director, Medical Programs, Office of the Assistant Secretary of Defense for Nuclear, Chemical, and Biologic Defense Programs, US Department of Defense; Stefanie Li Yee Kam, MSc, MA, Researcher, S. Rajaratnam School of International Studies, Nanyang Technological University, Singapore; Hussein Omar Khan, MBA, MSc, Chief Assistant Director, DNA Databank Division, Criminal Investigation Department, Royal Malaysia Police; Krishna Khanal, MSc, MBA, Research Analyst, International Center for Political Violence and Terrorism Research; S. Rajaratnam, School of International Studies, Nanyang Technological University, Singapore; Kwa Chong Guan, MA, Senior Fellow, S. Rajaratnam School of International Studies, Nanyang Technological University, Singapore; James W. Le Duc, PhD, Director, Galveston National Laboratory; Professor, Microbiology and Immunology; John Sealy Distinguished Chair in Tropical and Emerging Virology, University of Texas Medical Branch, USA; Lee Fook Kay, PhD, Chief Science and Technology Officer, Ministry of Home Affairs,

Singapore New Phoenix Park; Vernon Lee, MBBS, PhD, MPH, MBA, FAMS, Head, Singapore Armed Forces Biodefense Center; Irma R. Makalinao, MD, MA, FPPS, FPSCOT, Professor, Department of Pharmacology and Toxicology, University of the Philippines Manila College of Medicine; Michael S. Malley, PhD, Lecturer, Naval Postgraduate School, USA; Diane Meyer, RN, MPH, Senior Analyst, Johns Hopkins Center for Health Security, USA: His Excellency Ashok Kumar Mirpuri, MA, Singapore's Ambassador to the United States of America; Amanda Moodie, MA, Research Analyst, Center for the Study of Weapons of Mass Destruction, National Defense University, USA; Nazalan Najimudin, PhD, Professor (Molecular Genetics), School of Biologic Sciences, Universiti Sains Malaysia; Ng Lee Ching, PhD, Director, Environmental Health Institute, Singapore Environment Institute; Ambassador Ong Keng Yong, Executive Deputy Chairman, S. Rajaratnam School of International Studies, Nanyang Technological University; Director, Institute of Defence and Strategic Studies, Singapore; Graham Ong-Webb, PhD, S. Rajaratnam School of International Studies, Nanyang Technological University, Singapore; Ooi Peng Lim Steven, MBBS, MSc, MPH, FAMS; Deputy Director (Policy and Control), Communicable Diseases Division; Adjunct Programme Director, Preventive Medicine Residency Programme, Ministry of Health, Singapore; Tara O'Toole, MD, MPH, Former Undersecretary for Science and Technology, US Department of Homeland Security; Imran Pambudi, MD, Deputy Director, Multilateral Health Cooperation, Ministry of Health, Indonesia; Tikki Elka Pangestu, PhD, Visiting Professor, Lee Kuan Yew School of Public Policy, National University of Singapore; Sumi Parenjape, PhD, MPH, Director, Technology Innovation, Vulcan Inc., USA; Nakorn Premsri, MD, Director of Bureau of Epidemiology, Department of Disease Control, Ministry of Public Health, Thailand; MG Ben Rimba, MD, MHA, Military Medicine, Indonesian Defense Force; Sanjana Ravi, MPH, Senior Analyst, Johns Hopkins Center for Health Security, USA; John C. Schaefer III, Malaysia and Indonesia Country Manager, Cooperative Biologic Engagement Program, Defense Threat Reduction Agency, US Department of Defense; Matthew P. Shearer, MPH, Senior Analyst, Johns Hopkins Center for Health Security, USA; Siswanto, MD, MHP, DTM, Director, Center for Applied Health Technology and Clinical Epidemiology, National Institute of Health Research and Development, Ministry of Health, Indonesia; Ratna Sitompul, MD, PhD, Department of Ophthalmology, Faculty of Medicine, Universitas Indonesia; Amin Soebandrio, PhD, Professor, Medical Faculty, University of Indonesia; Angkana Sommanustweechai, DVM, International Health Policy Program,

Ministry of Public Health, Thailand; Subramaniam Sathasivam, MD, Minister of Health, Malaysia; Pratiwi Pujilestari Sudarmono, MD, PhD, Professor of Clinical Microbiology, Faculty of Medicine, Universitas Indonesia; Lokman Hakim bin Sulaiman, MPH, MD, PhD, Deputy Director General of Health (Public Health), Ministry of Health, Malaysia; Kitpong Sunchatawirul, MD, Bamrasnaradura Infectious Disease Institute, Department of Disease Control, Ministry of Public Health, Thailand; Daniel Tjen, MD, SpS, Chief Medical Officer, Mayapada Healthcare Group, Indonesia; R. Emerson Tuttle, DVM, MA, Regional Science Manager, Cooperative Biologic Engagement Program, Defense Threat Reduction Agency, US Department of Defense; Viji Vijayan, MBBS, MSc, MD, Director, Research Operations and Department of Safety, Health and Environment, Duke–National University of Singapore Graduate Medical School, Singapore; Kyaw San Wai, MSc, Senior Analyst, S. Rajaratnam School of International Studies, Nanyang Technological University, Singapore; Linfa Wang, PhD, FTSE, Professor and Director, Programme in Emerging Infectious Diseases, Duke–National University of Singapore Graduate Medical School, Singapore; Andrew Weber, MSFS, Assistant Secretary of Defense for Nuclear, Chemical, and Biologic Defense Programs, US Department of Defense; Suwit Wibulpolprasert, MD, Senior Advisor, Ministry of Public Health, Thailand; Annelies Wilder-Smith, MD, PhD, DTM&H, MIH, FAMS, FACTM, Professor of Infectious Diseases Research; Lead, Global Health and Vaccinology, Lee Kong Chian School of Medicine, Nanyang Technological University, Singapore; Michelle Yap, Senior Assistant Director, Strategic Technical Engagement and International Liaison, Office of the Chief Science and Technology Officer, Ministry of Home Affairs, Singapore; Jaime Yassif, PhD, Program Officer, Biosecurity and Pandemic Preparedness, Open Philanthropy Project, USA; Zalini Binti Yunus, PhD, Senior Director, Biologic and Toxin Weapons Convention Nucleus, Science and Technology Research Institute for Defence, Ministry of Defence, Malaysia.
